# Patient Referral and Education Program Prior to Renal Replacement Therapy (PREP-RRT): A Pilot Study

**DOI:** 10.1007/s11606-025-09699-w

**Published:** 2025-07-08

**Authors:** Milda R. Saunders, Lindsay Zasadzinski, Cynthia Gaspard, Adewumi Omoniyi, Akilah King, James P. Lash, Monica E. Peek

**Affiliations:** 1 General Internal Medicine, University of Chicago Medicine, Chicago, IL USA; 2 Division of Nephrology, Department of Medicine University of Illinois Chicago, Chicago, USA

## Abstract

**Background:**

African American individuals with CKD are less likely to receive early CKD education and care than their White counterparts and have a more rapid progression to kidney failure.

**Objective:**

To determine if a brief inpatient intervention increased CKD knowledge.

**Design:**

Pre-post evaluation of a pilot intervention.

**Participants:**

The PREP-RRT pilot study recruited hospitalized adult African American patients with an estimated glomerular filtration rate (GFR) ≤ 45 from a general medicine inpatient service at a Midwestern academic medical center.

**Interventions:**

A social worker provided CKD patient education and motivational interviewing for behavior change.

**Main Measures:**

The primary outcome was change in patient knowledge about CKD, using the Kidney Knowledge Survey (KiKS), and kidney failure treatment options*.*

**Key Results:**

Out of the 60 patients, 52% were female (*n* = 21) with a mean age of 61 years old (range 28–76). Only 45% of patients (*n* = 27) had sufficient CKD knowledge at baseline. The median pre-intervention CKD knowledge score was 16 (interquartile range (IQR) 13–18) and the post-intervention score was 19 (IQR 17–21). Paired *t*-tests showed significant improvement in the primary outcome, CKD knowledge (*p* < 0.001). Overall baseline knowledge of kidney failure treatment modalities was low (< 30% with some to great knowledge for all modalities), and participants reported a significant increase in knowledge across kidney failure treatment modalities (all *p* < 0.05 except *p* = 0.17 conservative management). Patient intent to make lifestyle changes was high pre-intervention and did not change significantly.

**Conclusions:**

A brief, culturally tailored inpatient intervention successfully increased knowledge of CKD and kidney failure treatment options for African American hospitalized patients. More intensive interventions are needed to sustain knowledge gains and motivation for lifestyle changes.

**Supplementary Information:**

The online version contains supplementary material available at 10.1007/s11606-025-09699-w.

## INTRODUCTION

Chronic kidney disease (CKD) affects approximately 15% of US adults.^1^ Individuals with CKD experience high morbidity, mortality, and increased health care costs both as a direct effect of CKD and from complications of CKD co-morbid conditions like diabetes, hypertension, and cardiovascular disease.^1–3^ Patient education about CKD and CKD self-management allows individuals to manage risk factors to prevent progression to kidney failure or end-stage kidney disease (ESKD). Many patients with CKD do not receive guideline-recommended education, and patients with advanced CKD are often not aware of their CKD diagnosis.^4^ Patient education prior to renal replacement therapy (RRT) delays progression of kidney failure and leads to patient-preferred RRT choices including pre-emptive transplant, home dialysis, and access placement.^5–7^

African American patients are particularly at risk for late CKD detection and insufficient education. African American individuals with CKD are less likely to receive early CKD education and care than their White counterparts and have a more rapid progression to kidney failure.^1,8^ African American and other racially minoritized patients are less likely to be seen by a nephrologist prior to dialysis initiation.^5^ Patients report limited time for decision-making due to urgent initiation of dialysis as well as limited awareness and understanding of other RRT modalities.^9,10^

The hospital is an important, often overlooked, location for patient education and linkage to nephrology care for patients with advanced CKD. Many patients with advanced CKD are hospitalized within 3 months of dialysis initiation, which represents an opportunity to identify existing CKD and to use a multidisciplinary approach to preventive care, patient education, and patient-provider planning for future RRT needs.^11^ Patients may be more receptive to education efforts due to their concerns about their acute illness.^12,13^ Finally, many patients who have difficulty accessing outpatient care often appear in an inpatient setting. One strategy to address African Americans’ reduced access to pre-RRT education and needs is to provide both education and linkage to outpatient care during inpatient hospitalizations.

We developed and tested the Patient Referral and Education Program prior to Renal Replacement Therapy (PREP-RRT), a pilot intervention to educate and refer hospitalized African American patients who may not be well linked to the medical system. We sought to determine whether a socio-culturally tailored intervention, combining patient education and motivational interviewing, could reduce knowledge gaps related to CKD and RRT and increase patient intent to make lifestyle changes.

## METHODS

### Recruitment and Patient Participation

The PREP-RRT study was conducted in the general medicine inpatient service at a Midwestern urban academic medical center. Participants were hospitalized, English-speaking, African American patients, between the ages of 18 and 75 years old with an estimated glomerular filtration rate (GFR) ≤ 45, who were not on dialysis or post-kidney transplant, and without significant cognitive impairment. Participants were recruited between 3/2018 and 5/2019. We developed a protocol to estimate baseline CKD stage using current inpatient values as well as prior inpatient and outpatient values, if available. Individuals were excluded if they were not able to consent or could not communicate in English. For more information, see the full study protocol.^14^

This study was a sub-study of the Hospitalist Project, an ongoing clinical study that examines a variety of outcomes of general medicine patients hospitalized at our institution, and includes administrative data, inpatient interviews, and a 30-day follow-up by phone.^15^ The Hospitalist Project staff obtained basic demographic information and medical history. All inpatients recruited to the Hospitalist Project were screened daily by the Research Assistant (RA) to identify patient eligibility for the PREP-RRT study. We obtained all appropriate ethical and institutional review board approvals.

### Study Design

PREP-RRT is an inpatient pilot intervention. The evaluation of the PREP-RRT study was measured by using pre- and post-test questionnaires administered by the RA to assess patient understanding of their health and kidney disease, and kidney failure treatment knowledge and preferences. After screening and identifying appropriate patients, the RA acquired verbal consent. There were three phases to the study. All took place within the patient’s private room. The first phase consisted of an RA-facilitated baseline survey about CKD and RRT knowledge and other patient characteristics. Participants who did not need assistance completed the baseline survey on their own. For participants with low literacy or poor vision, the RA read the questions and recorded their answers. The RA then gave the participant kidney health education materials and contacted the patient educator, a social worker with nephrology expertise and training in health education.

In the second phase, the patient educator led a semi-structured, in-person education session using culturally-tailored education materials that focus on CKD awareness, CKD self-care, and RRT planning. The 19-page booklet reviews the function of kidneys in the body; the definition and cause of chronic kidney disease; measuring kidney function; how to manage diet, fluid, and kidney treatment; CKD stage-specific self-care; kidney failure treatment options including hemodialysis, peritoneal dialysis, transplant, dialysis access, and conservative management; socio-economic barriers to access and care; and supportive resources.

In addition to providing CKD education, the patient educator incorporated motivational interviewing (MI) to stimulate the patient’s commitment to action to improve their CKD self-management, interaction with the medical system, and commitment to RRT selection. The patient educator contacted the RA after completing the education session. The education session lasted between 20 and 45 min, depending on participant baseline knowledge, interest, and perceived barriers. In the third and final phase, the RA provided the participant with the post-intervention survey. After completion of all three phases, participants received a $25 gift card.

### Measures and Outcomes

#### Primary Outcome: Change in Patient Knowledge about CKD and Kidney Failure Treatment

To assess knowledge of kidney disease, we used the validated Kidney Knowledge Survey (KiKS) and determined sufficient knowledge to be > 66% questions correct on the KiKS.^16^ We used an investigator-developed instrument to assess knowledge and preferences about kidney failure treatment options.^17^ The outcome variable was created based on whether a participant’s post-survey score was a standard deviation above the mean baseline kidney disease knowledge.^16^

#### Secondary Outcome: Motivation and Intent

Investigator-developed instruments were used to measure patient intent to make lifestyle changes.^17^ Questions included intent to change dietary habits, physical activity, medication adherence, and management of comorbid conditions (e.g., hypertension) to slow the progression of CKD. Patients were asked how likely they are to make changes to diet, exercise regimen, and kidney care over the next 3 months. The 5-point Likert scale ranged from extremely unlikely, unlikely, not sure, likely, to extremely likely.

#### Covariates

Patient information collected at baseline included patient age, gender, health literacy, perceived efficacy, and self-report of comorbid conditions. We measured health literacy using the Single-Item Literacy Screener (SILS).^18^ The question: “How confident are you filling out medical forms by yourself?” Individuals answering *somewhat, a little bit, or not at all* are classified as having inadequate health literacy. We abstracted the following clinical information from the electronic medical record (EMR): eGFR (to classify CKD stage), body mass index (BMI), CKD-related comorbidities (i.e., diabetes (DM), hypertension (HTM), congestive heart failure (CHF), and coronary heart disease (CAD)), as well as information about prior visits to nephrology (outside and within our institution) and primary care visits at our institution.

### Data Analysis

Analyses were performed using RStudio software.^19^ Descriptive statistics were used to summarize patient sociodemographic characteristics, health literacy, and self-efficacy at baseline. We measured change in kidney disease knowledge through a paired *t*-test of knowledge scores pre- and post-intervention. McNemar tests were used to compare responses pre- and post-intervention for knowledge of kidney failure treatment options, awareness of own CKD-related co-morbidities, and intent to make lifestyle changes. To determine factors associated with baseline as well as improvement in kidney disease knowledge, bivariable and multivariable logistic regression analyses were conducted, adjusting for the following covariates: age, gender, CKD stage, health literacy, and connection to outpatient care.

## RESULTS

We screened hospitalist project patients and identified 124 eligible subjects. We approached 107 patients. Of those, 86 consented and completed the pre-survey, and 68 completed all phases. From the completed intervention group, eight participants were excluded—one due to missing data and seven for not meeting study criteria. Of those who did not meet study criteria, two patients were excluded because they had a prior kidney transplant (*n* = 2) and the others had improvement in kidney function after hospital discharge, eGFR > 60 (*n* = 5)—leaving a total of 60 eligible participants.

Of the 60 patients, 31 were female (52%) (Table 1). The mean age of the study population was 61 years (range 28–76) and seven (11.7%) participants had stage 3 A CKD, 28 (46.7%) had stage 3B, 12 (20%) had stage 4, and 13 (21.7%) had stage 5 CKD. Participants’ co-morbidities included HTN (90%), DM (66.7%), CHF (45%), and CAD (36.7%). About 26.7% of patients had had inadequate health literacy.

**Table 1:** Patient Sociodemographic and Clinical Characteristics

Age, mean (range)	61.2 (28, 76)
BMI, mean (range)	34.39 (17, 72)
Gender, female	31 (51.7%)
Married	10 (16.7%)
Health literacy: confident with forms	
Adequate: extremely/quite a bit	44 (73.3%)
Inadequate: somewhat or less	16 (26.7%)
CKD stage	
3A	7 (11.7%)
3B	28 (46.7%)
4	12 (20%)
5	13 (21.7%)
Diabetes	40 (66.7%)
Congestive heart failure	27 (45%)
Coronary artery disease	22 (36.7%)
Hypertension	54 (90%)
Insurance	
Medicaid	29 (48.3%)
Medicare	14 (23.3%)
Private	4 (6.7%)
Previous nephrology appointment*	15 (25%)
UChicago primary doctor	22 (36.7%)
Health care providers patients reported seeing in prior 12 months**	
Primary care doctor (e.g., general internist, family practice)	48 (80%)
Heart doctor (cardiologist)	29 (48.3%)
Diabetes doctor (endocrinologist)	32 (53.3%)
Kidney doctor (nephrologist)	27 (45%)

^*^Per EMR review

^**^By patient self-report

**Figure 1:**
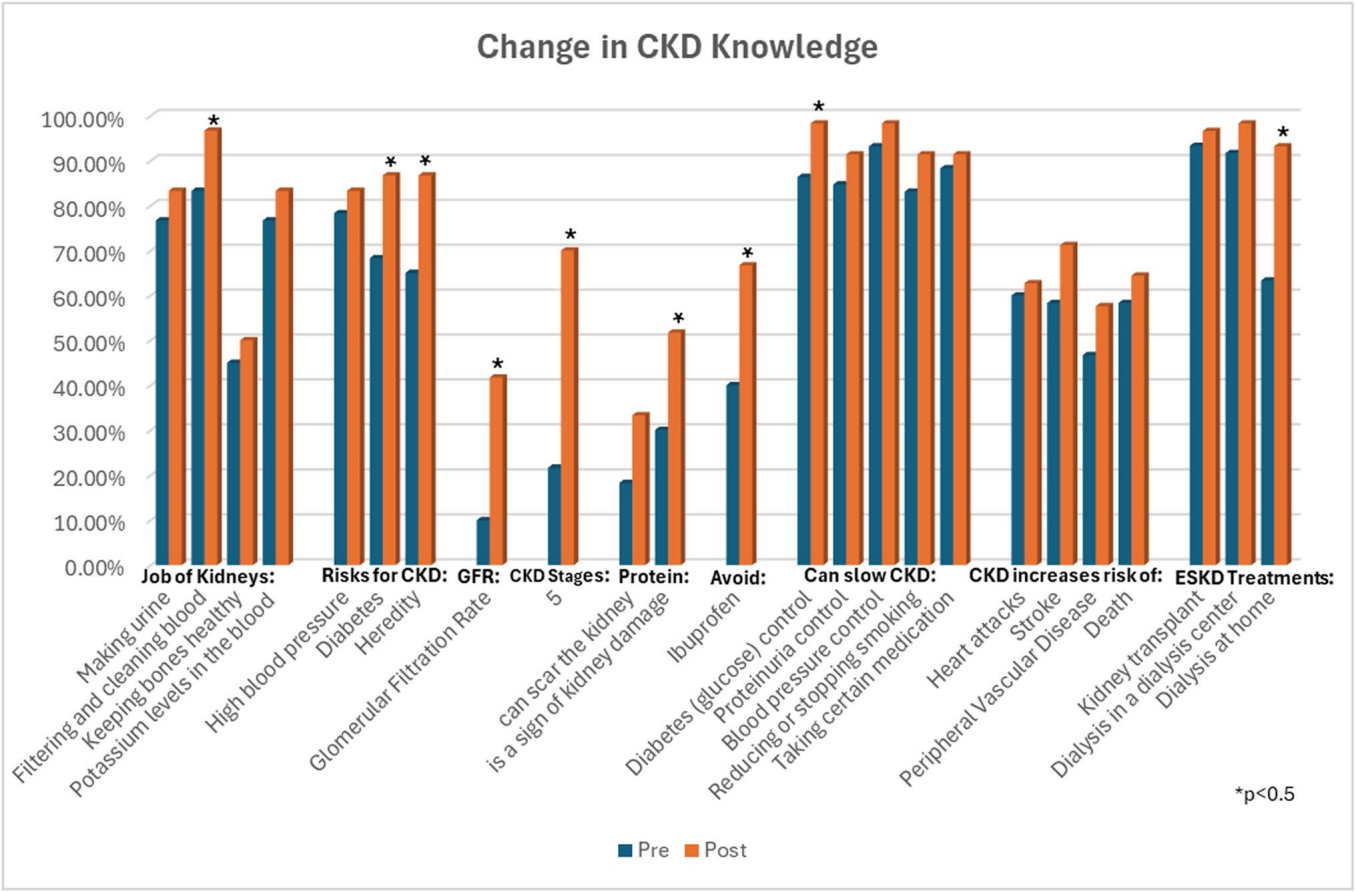
**Change in CKD knowledge by question.**

Paired *t*-tests showed significant improvement in the primary outcome, CKD knowledge (*p* < 0.001). The median pre-intervention CKD knowledge score was 16 (interquartile range (IQR) 13–18) and post-intervention score was 19 (IQR 17–21) out of 25 questions (Appendix Table 1). Overall, patients had high knowledge regarding actions that slow CKD progression, with over 80% scoring correctly on all questions in this category in both the pre- and post-survey (Fig. 1). At baseline, participants had low knowledge (< 50%) on several topics including GFR, CKD stages, proteinuria, and medications to avoid. Most of these areas saw statistically significant improvement post-intervention. In addition, the post-intervention survey revealed a significantly greater proportion of participants identifying home dialysis as an RRT option.

We found that 45% of patients (*n* = 27) had sufficient CKD knowledge at baseline, defined as > 66% correct. In bivariable analysis, having had a previous nephrology appointment, adequate health literacy, and stage 5 CKD were associated with an increased likelihood of having adequate baseline knowledge (Table 2). Participants with CKD stage 5 had greater odds of having adequate baseline knowledge compared to those with stage 3 (OR 8.25, 95% CI 1.86, 58.83); those with a previous nephrology appointment had greater odds of having baseline CKD knowledge compared to those without (OR 4.98, 95% CI 1.45, 20.42) and those with inadequate health literacy had lower odds of baseline knowledge compared to those with adequate (OR 0.05, 95% CI 0.00, 0.26). In a multi-variable model, only health literacy was a significant predictor of baseline knowledge. When examining post-intervention increase in CKD knowledge (Table 3), having CKD stage 5 was the only factor associated with greater likelihood of improved knowledge post-intervention in the bivariable and multi-variable models (OR 5.0, 95% CI 1.27, 25.38; and OR 4.76, 95% CI 1.06, 27.04, respectively).

**Table 2:** Factors Associated with Sufficient Kidney Disease Knowledge at Baseline

	Crude OR		Adjusted OR	
	Odds ratio (95% CI)	*p*-value	Odds ratio (95% CI)	*p*-value
Age	0.98 (0.93–1.03)	0.43	0.99 (0.93–1.07)	0.88
Gender, male	1.29 (0.47–3.62)	0.62	0.93 (0.21–3.93)	0.92
Health literacy, inadequate	0.05 (0.00–0.26)	0.004**	0.06 (0.00–0.37)	0.01*
CKD stage 4	0.30 (0.04–1.36)	0.16	0.17 (0.02–0.99)	0.07
CKD stage 5	8.25 (1.86–58.83)	0.01*	2.61 (0.44–21.73)	0.32
Previous nephrology connection	4.98 (1.45–20.42)	0.02*	2.45 (0.49–13.56)	0.28
Married	2.00 (0.50–8.83)	0.33	–-	–-
UChicago primary doctor	2.48 (0.86–7.47)	0.10	–-	–-
Insurance, Medicare/private	2.23 (0.68–7.69)	0.19	–-	–-
Diabetes	2.58 (0.85–8.55)	0.10	–-	–-
Congestive heart failure	1.66 (0.59–4.71)	0.34	–-	–-
Coronary artery disease	0.77 (0.26–2.21)	0.63	–-	–-
Hypertension	0.37 (0.05–2.07)	0.28	–-	–-

Sufficient knowledge ≥ 66% correct

^*^p < 0.05; **p < 0.01;

^a^Reference is CKD stage 3

**Table 3:** Factors Associated with Post-Intervention Improvement in Kidney Disease Knowledge

Predictor	Crude OR		Adjusted OR	
	Odds ratio (95% CI)	*p*-value	Odds ratio (95% CI)	*p*-value
Age	0.97 (0.91–1.02)	0.21	0.98 (0.92–1.05)	0.59
Gender, male	1.70 (0.61–4.87)	0.32	1.39 (0.41–4.80)	0.59
Health literacy, inadequate	0.55 (0.15–1.77)	0.33	0.86 (0.21–3.38)	0.84
CKD stage 4	0.14 (0.01–0.82)	0.07	0.15 (0.01–0.98)	0.09
CKD stage 5	5.00 (1.27–25.38)	0.03*	4.76 (1.06–27.0)	0.05
Married	0.98 (0.22–3.96)	0.98	–-	–-
Insurance, Medicare/Private	1.21 (0.35–4.10)	0.76	–-	–-
Diabetes	3.00 (0.96–10.68)	0.07	–-	–-
congestive heart failure	0.71 (0.25–1.98)	0.51	–-	–-
coronary artery disease	0.38 (0.11–1.13)	0.09	–-	–-
hypertension	0.32 (0.04–1.78)	0.21	–-	–-
Previous nephrology connection	1.88 (0.58–6.29)	0.29	–-	–-
UChicago primary doctor	0.52 (0.17–1.52)	0.24	–-	–-

^*^p < 0.05; **p < 0.01;

Overall knowledge of all treatment modalities was low (< 30% reporting some to great knowledge) at baseline (Table 4). Participants reported they were most knowledgeable about living donor transplantation (28.3%) and dialysis in dialysis centers (28.3%) and least knowledgeable about peritoneal dialysis (10%). Participants reported a significant increase in knowledge across all ESKD treatment modalities except for conservative management (all *p* < 0.05 except *p* = 0.17 conservative management).

**Table 4:** Self-Reported Knowledge of Kidney Failure Treatment Options

Survey item	Pre-survey	Post-survey	*p*-value
Dialysis in a dialysis center	17 (28.3%)	30 (50%)	0.009**
Dialysis at home	15 (25%)	25 (41.7%)	0.04*
Peritoneal dialysis	6 (10%)	21 (35%)	< 0.001***
Kidney transplant with a deceased donor^*1*^	13 (21.7%)	26 (44.1%)	0.009**
Kidney transplant with a living donor	17 (28.3%)	34 (56.7%)	0.001**
Symptom management only (palliative or conservative treatment) for kidney failure	13 (21.7%)	20 (33.3%)	0.17

^*^p < 0.05; **p < 0.01; ***p < 0.001

^1^Post-survey, *N* = 59

We also examined their intent to make lifestyle changes and understanding of their health conditions. A large proportion of patients reported they planned to make positive lifestyle changes. At baseline, more than 95% of participants reported wanting to address blood pressure (100%), diabetes (98.3%), CKD treatment knowledge (100%), and kidney failure treatment knowledge (98.3%). At baseline, < 75% of participants reported a strong likelihood of obtaining dialysis access or making an appointment at a transplant center. Post-intervention, there was no statistically significant difference (Appendix Table 2). Only 27% of participants reported being likely or extremely likely to have kidney failure in the next 5 years, while over 40% reported uncertainty (Appendix Table 3). There were no significant changes in risk perception post-intervention. There was no association between CKD stage and reporting a high likelihood of kidney failure (χ^2^ = 1.20, p = 0.55; analysis not shown).

## DISCUSSION

Overall, the Patient Referral and Education Program prior to Renal Replacement Therapy (PREP-RRT) demonstrates that a culturally tailored, hospital-based intervention is feasible and effective in increasing knowledge about CKD and kidney failure treatment modalities for African American patients. After our brief inpatient intervention, we found significant improvement in knowledge about CKD risk factors, CKD stages, and treatments for kidney failure.

PREP-RRT and similar interventions are needed to better inform African American patients with advanced CKD about their disease, its management, and kidney failure treatment options. Effective patient education is of critical importance since African American patients have a higher prevalence of kidney failure and more rapid progression from CKD to kidney failure, and have reduced access to effective RRT treatments.^20–23^

We found that many participants had low baseline CKD knowledge, little information about RRT options, and inadequate health literacy. Previous work has also found that patients in a general nephrology clinic had low CKD-specific knowledge, and scores on the Kidney Disease Knowledge Survey ranged from 66 to 70%.^16,24,25^ Our participants had a mean score of 60% at baseline and improved to 74% after the intervention. CKD knowledge about GFR, CKD stages, medications to avoid, proteinuria, and treatment modalities was low before the intervention but improved significantly, which demonstrates that our intervention was effective in improving knowledge in areas where information was most needed. Prior work revealed that CKD patient education may lack information about modality options and decision support, resulting in patients not feeling adequately prepared to choose between kidney failure treatment options.^26–28^

We also found that over a quarter (26.7%) of participants had inadequate health literacy, similar to prior work that reported limited health literacy in 25% of CKD patients.^29^ Inadequate health literacy was an important predictor of both low baseline CKD knowledge and lower likelihood of improved CKD knowledge post-intervention. Future iterations of this intervention should focus on tools to better reach low-literacy patients.

We also found that patients with CKD stage 5 were more likely to have adequate kidney disease knowledge at baseline and improve their knowledge after the intervention when compared to stage 3 CKD. Patients with later stage CKD often have higher rates of hospitalizations and more nephrology visits, which may increase both the salience of and opportunities for CKD education.^30,31^ In addition, as CKD progresses, increasing symptoms and decreasing quality of life may motivate patients to learn more about CKD in order to better manage their disease.^32^ Finally, we found that prior nephrology visits were associated with increased likelihood of adequate baseline CKD knowledge.

This study’s design contains multiple strengths. First, the study focuses on African American patients and uses culturally tailored education materials and a racially concordant research team. In addition, the study recruits and educates patients while they are hospitalized. The hospital as the site of intervention is innovative and may help to reduce disparities because many patients who have difficulty accessing outpatient care often appear in an acute care setting.^11^ While many hospital-based interventions educate patients about the acute issue associated with their hospitalization,^33,34^ we used hospitalization as an opportunity to screen and educate patients about their CKD, an issue often not directly related to their admission. CKD education within a hospital setting is novel in the USA. Outside the USA, patients may receive care in inpatient kidney units where CKD-specific education is more common.^35^ Another study strength is the use of motivational interviewing (MI) to support patients in resolving ambivalence and developing autonomy in the decisions regarding lifestyle changes and medical care. CKD education in conjunction with MI enables patients to make informed decisions and assume a collaborative role in their treatment with medical providers.

Our study limitations include a small single-site sample without a control group, which limits generalizability. In addition, we had difficulties ascertaining “true” CKD stages due to fluctuations in eGFR in the setting of a hospitalization. Despite screening patients who had inpatient eGFR < 45, 7% of individuals had eGFR recovery post-discharge and were subsequently excluded. Prior work revealed patients hospitalized at our institution have high rates of acute kidney injury (AKI), which is a risk factor for CKD.^36,37^ While these individuals might also benefit from CKD education, they were outside the focus of our study. Furthermore, the patient educator was an African American social worker with an outgoing personality, trained in MI, and knowledgeable about CKD. The combination of attributes that made her an asset to the project may also make the intervention difficult to replicate in other settings or to scale up within our own institution. Finally, we do not know how long patients’ knowledge gain persisted post-discharge.

Our brief hospital-based intervention did not improve patient intent to make lifestyle changes or kidney failure treatment choices. There was no significant difference between intent pre- and post-intervention, likely due to the high number of patients who reported intent to change at baseline. We were surprised that so many patients intended to make lifestyle changes given that evidence shows that many patients often do not. Future interventions should focus not on increasing intent to change lifestyle, but on ways to sustain motivation over time and to offer actionable steps to implement those changes. Our future work will focus on testing the intervention in a randomized fashion using a larger sample size and a control group. The next iteration will have a more intensive focus on post-discharge behavior change as well as a longer follow-up to assess sustained knowledge gains and behavioral change over a longer period. We also plan to expand intervention accessibility by developing and testing a culturally tailored online intervention to provide a less staff-intensive mode for patient education.

## CONCLUSION

African Americans progress more rapidly to kidney failure and may receive less information about kidney failure treatment options compared to their White counterparts. As a result, many African American patients with advanced CKD may lack information about CKD, transplantation, and home dialysis modalities. The PREP RRT intervention was successful in educating patients on CKD, CKD self-care, and kidney failure treatment modalities.

## Supplementary Information

Below is the link to the electronic supplementary material.

ESM 1 (DOCX 25.6 KB)

## Data Availability

Data available upon request.
