# Supplementary material for: Patient Referral and Education Program Prior to Renal Replacement Therapy (PREP-RRT): A Pilot Study
Source: J Gen Intern Med. 2025 Jul 8;41(1):111–8. doi: 10.1007/s11606-025-09699-w (PMC12855683; doi:10.1007/s11606-025-09699-w)
Supplement: Supplementary file 1 — (DOCX 25.6 KB) [file 11606_2025_9699_MOESM1_ESM.docx]

*Appendix*

*Appendix Table 1: Kidney Disease Knowledge Survey Pre and Post Scores*

|  | Pre-Survey | Post-Survey | P-value |
| --- | --- | --- | --- |
| **Important jobs of the kidney include*...** |  | | |
| Making urine | 46 (76.7%) | 50 (83.3%) | .22 |
| Filtering and cleaning blood | 50 (83.3%) | 58 (96.7%) | .03* |
| Keeping bones healthy | 27 (45%) | 30 (50%) | .66 |
| Keeping potassium levels in the blood normal | 46 (76.7%) | 50 (83.3%) | .42 |
| **Causes of Chronic Kidney Disease include*...** |  | | |
| High blood pressure | 47 (78.3%) | 50 (83.3%) | .58 |
| Diabetes | 41 (68.3%) | 52 (86.7%) | .03* |
| Heredity | 39 (65%) | 52 (86.7%) | .006** |
| **"GFR" stands for...** |  | | |
| Glomerular filtration rate | 6 (10%) | 25 (41.7%) | < .001*** |
| **The number of CKD stages is...** |  | | |
| 5 | 13 (21.7%) | 42 (70%) | < .001*** |
| **Too much protein is bad for the kidney because*...** |  | | |
| It can scar the kidney | 11 (18.3%) | 20 (33.3%) | .07 |
| It is a sign of kidney damage | 18 (30%) | 31 (51.7%) | .01* |
| **One medication CKD patients should avoid taking is...** |  | | |
| Ibuprofen | 24 (40%) | 40 (66.7%) | < .001*** |
| **Actions that can slow the progression of CKD include*...***^1^* |  | | |
| Diabetes (glucose) control | 51 (86.4%) | 57 (98.3%) | .02* |
| Proteinuria control | 50 (84.7%) | 53 (91.4%) | .55 |
| Blood pressure control | 55 (93.2%) | 57 (98.3%) | .37 |
| Reducing or stopping smoking | 49 (83.1%) | 53 (91.4%) | .18 |
| Taking certain medication | 53 (88.3%) | 53 (91.4%) | .77 |
| **CKD increases the risk for*...***^2^* |  | | |
| Heart attacks | 36 (60%) | 37 (62.7%) | > .99 |
| Stroke | 35 (58.3%) | 42 (71.2%) | .10 |
| Peripheral Vascular Disease | 28 (46.7%) | 34 (57.6%) | .15 |
| Death | 35 (58.3%) | 38 (64.4%) | .34 |
| **Possible treatments for CKD include*...***^2^* |  | | |
| Kidney transplant | 56 (93.3%) | 57 (96.6%) | .62 |
| Dialysis in a dialysis center | 55 (91.7%) | 58 (98.3%) | .13 |
| Dialysis at home | 38 (63.3%) | 55 (93.2%) | < .001*** |
| Overall score of Correct Answers, Median (Min-Max) | 16 (5-22) | 19 (9-25) | < .001*** |

*^1^*Post-Survey, N = 58., *^2^*Post-Survey, N = 59., *Can select all that apply

*Appendix Table 2: McNemar’s Analysis of Pre and Post Differences Regarding Intent to Make Lifestyle Changes (N = 60)*

| Survey Item | Pre-Survey | Post-Survey | P-Value |
| --- | --- | --- | --- |
| Make changes to diet | 56 (93.3%) | 57 (95%) | > .99 |
| Increase physical activity | 57 (95%) | 57 (95%) | > .99 |
| Work to control blood pressure | 60 (100%) | 60 (100%) | > .99 |
| Work to control diabetes*^1^* | 59 (98.3%) | 59 (100%) | > .99 |
| Stop or decrease smoking*^1^* | 57 (95%) | 57 (96.6%) | > .99 |
| See a kidney doctor*^2^* | 57 (95%) | 55 (96.5%) | > .99 |
| Learn more about CKD treatments | 60 (100%) | 59 (98.3%) | > .99 |
| Discuss kidney disease with family and friends | 55 (91.7%) | 54 (90%) | > .99 |
| Learn more about treatments for kidney failure | 59 (98.3%) | 59 (98.3%) | > .99 |
| Choose a treatment if kidneys failed*^3^* | 54 (93.1%) | 58 (98.3%) | .37 |
| Get dialysis access for peritoneal dialysis/hemodialysis*^1^* | 40 (66.7%) | 38 (64.4%) | > .99 |
| Make an appointment at the transplant center*^1^* | 43 (71.7%) | 36 (61%) | .29 |
| Talk to family about living donation*^4^* | 49 (81.7%) | 51 (87.9%) | .29 |

*^1^*Post-Survey, N = 59.

*^2^*Post-Survey, N = 57.

*^3^*Pre-Survey, N = 58. Post-Survey, N = 59.

*^4^*Post-Survey, N = 58.

*Appendix Table 3: McNemar’s Analysis of Pre and Post Differences Regarding Confidence in Health Understanding (N = 60)*

| Survey Item | Pre-Survey | Post-Survey | P-value |
| --- | --- | --- | --- |
| *Which of the following conditions has a healthcare provider told you that you have? (n% of correct answers - yes or no)* | | | |
| Diabetes*^1^* | 53 (91.4%) | 49 (89.1%) | .48 |
| High Blood Pressure or Hypertension*^2^* | 58 (96.7%) | 54 (96.4%) | > .99 |
| Obesity*^3^* | 40 (66.7%) | 36 (67.9%) | > .99 |
| Kidney Disease*^4^* | 34 (59.6%) | 34 (63%) | > .99 |
| *How likely do you think it is that your kidneys will stop working (you will go on to have kidney failure) in the next 5 years?^5^* | | | |
| Extremely unlikely | 16 (26.7%) | 14 (25.5%) | > .99 |
| Mostly unlikely | 4 (6.7%) | 4 (7.3%) | > .99 |
| Not sure | 24 (40%) | 23 (41.8%) | > .99 |
| Mostly likely | 7 (11.7%) | 6 (10.9%) | > .99 |
| Extremely likely | 9 (15%) | 8 (14.5%) | > .99 |
| Already had kidney failure | 0 (0.0%) | 0 (0.0%) | > .99 |

*^1^*Pre-survey, N = 58. Post-survey, N = 55.

*^2^*Post-survey, N = 56.

*^3^*Post-survey, N = 53.

*^4^*Pre-survey, N = 57. Post-survey, N = 54.

*^5^*Post-survey, N = 55.
